# The intervention effect of exercise on the attention of patients with depression: a systematic review

**DOI:** 10.3389/fpsyg.2025.1536262

**Published:** 2025-04-28

**Authors:** Haijun Dong, Cong Liu, Man Qin

**Affiliations:** ^1^Department of Physical Education, University of Shanghai for Science and Technology, Shanghai, China; ^2^School of Physical Education, Shanghai University of Sport, Shanghai, China; ^3^School of Sports and Health, Shanghai Linxin Accounting and Finance University, Shanghai, China

**Keywords:** exercise, depression, attention, patients, a systematic review

## Abstract

**Objective:**

This paper aims to systematically evaluate the intervention effect of exercise on the attention of patients with depression.

**Methods:**

The randomized controlled trials of exercise intervention on the attention of patients with depression in six databases were retrieved by computer, and the quality of the included literature was evaluated by the PEDro scale. The meta-analysis, subgroup analysis, publication bias test, and sensitivity analysis were performed by Stata 17.0, and the quality of evidence was evaluated by GRADEpro.

**Results:**

A total of 11 literature involving 924 patients with depression were included. The results showed that exercise could improve the attention of patients with depression (Hedge’s *g* = 0.17, *p* = 0.01), exercise intensity (*p* = 0.00) had a regulatory effect on the intervention effect, and exercise form (*p* = 0.77), exercise duration (*p* = 0.58) and exercise cycle (*p* = 0.66) had no regulatory effect on the intervention effect.

**Conclusion:**

Exercise can improve the attention of patients with depression, among which moderate intensity has the best effect. This study has been registered on the international prospective register of systematic reviews Prospero (CRD4202477699).

## 1 Introduction

Depression is a common mental disease with persistent depression, decreased interest, and even pessimism. At present, there are nearly 100million patients with depression in China, and the lifetime prevalence rate of depression is 6.9% (Huang et al., 2019; Wang et al., 2022), and the World Health Organization has shown that depression will become the main killer of mankind in the 21st century. Patients with depression often exhibit cognitive impairments in executive function, information processing speed, memory, and attention (Kriesche et al., 2023). Most patients with depression find it hard to maintain sustained concentration and have abnormal attention selectivity and focus on negative things, ideas, and evaluations (Clegg et al., 2024). Attention and other cognitive impairments not only affect the efficiency of drug treatment for depression but also increase the risk of depression recurrence, causing a heavy burden on patients, families, and society (Porter and Douglas, 2019; Chinese Society of Psychiatry, 2020; Freedman et al., 2024).

Exercise has a positive effect on the prevention and treatment of depression, and it is also a non-drug means to improve cognitive function. A study investigated the effects of low-dose (4 kcal per kilogram of body weight per week) and high-dose (16 kcal per kilogram of body weight per week) exercise over a 12-week period on attention and visual memory in patients with depression. Attention and visual memory were assessed using the Big Circle/Little Circle and Pattern Recognition Memory tasks. The results demonstrated that the Mean Correct Latency of the Big Circle/Little Circle task was significantly reduced in both groups, while the Percent Correct of the Pattern Recognition Memory task significantly increased. These findings suggest that both doses of long-term exercise can improve attention and visual memory in patients with depression (Greer et al., 2015). Another study implemented a 16-week aerobic exercise intervention, consisting of at least three sessions per week, each lasting 30–60 min at moderate intensity, for patients with depression. Attention was evaluated using the Digit Span-Sequential Order and Continuous Performance Task. The results showed that the scores for both tasks significantly improved following the exercise intervention, indicating that 16 weeks of aerobic exercise can enhance attention in patients with depression (Chen et al., 2021). However, contradictory findings have also been reported. A systematic review, which included seven studies, concluded that exercise does not have a significant effect on improving attention (Brondino et al., 2017). Similarly, another study that incorporated six studies also indicated that exercise fails to improve attention (Sun et al., 2018). Moreover, meta-analyses have also examined the effects of long-term aerobic exercise on attention in individuals with depression. However, the findings similarly indicate that long-term aerobic exercise does not improve attentional function in patients with depression (Ren et al., 2024). In conclusion, whether exercise can improve the attention of patients with depression has not yet formed a consensus.

Previous studies show that although there are systematic reviews or meta-analyses to explore the intervention effect of exercise on the attention of patients with depression, there is no independent study to further explore the attention issue, such as not paying attention to the “dose–response relationship” between exercise factors and the attention of patients with depression. Based on this, this study will continue to explore the intervention effect of exercise on the attention of patients with depression, and further clarify the dose–response relationship of exercise factors on the intervention effect of attention, to find the best dose, to provide evidence for clinical practice and theoretical reference for researchers.

## 2 Research methods

The study followed the requirements of the Preferred Reporting Items for Systematic Reviews and meta analyses (Page et al., 2020) for data inclusion and statistical analysis. This study has been registered on the International Prospective Register of Systematic Reviews PROSPERO^1^ (No. CRD42023477699). The PRISMA 2020 checklist is attached.

The PICOS architecture of this study is shown in Table 1.

**Table 1:** PICOS framework of the effect of exercise on attention intervention in patients with depression.

PICOS	Details
Population	Patients with depression: meet any of the diagnostic criteria of the International Classification of Disease (ICD) and the Diagnostic and Statistical Manual of Mental Disorders (DSM)
Intervention	Exercise or exercise based on the intervention content of the control group
Comparison	Routine treatment, daily life, stretching exercise, etc.
Outcome	Relevant indicators of attention
Study design	Randomized controlled trial

### 2.1 Literature retrieval strategy

Two researchers (HJ D and C L) independently searched 6 databases including CNKI (China National Knowledge Internet), Wanfang, EMBASE, WOS, the Cochrane Library, and PubMed for randomized controlled trials on the effect of exercise on the attention of patients with depression. The retrieval date was from the establishment of each database to November 4, 2023. The method of combining subject words with free words is adopted, and the Boolean operation symbols “and” and “or” are used for combination connection, which is determined after repeated pre-inspection. If two researchers encounter differences, discuss and decide with the third researcher. The Chinese words were: 运动 (exercises), 体育锻炼 (physical exercises), 有氧运动 (aerobic exercises), 抗阻训练 (resistance exercises), 太极拳 (Tai Chi), 瑜伽 (Yoga), 八段锦 (Baduanjin), 快走 (speed walking), 慢跑 (jogging), 抑郁 (depression), 抑郁症 (depression disease), 抑郁症患者 (depressive patients), 认知功能 (cognitive function), 认知和注意力 (cognition and attention); the English words include: depression, depressive disorder, aerobic exercise, physical activity, yoga, Tai Chi, resistance training, cognitive performance, attention, etc. At the same time, it is supplemented by tracking the relevant systematic reviews and references included in the literature. The retrieval strategies of each database are shown in Table 2.

**Table 2:** Retrieval strategies of each database.

Database	Retrieval strategy
Cochrane and Pubmed	#1″Exercise”[Mesh] OR “Aerobic exercise” [Title/Abstract] OR “Resistance exercise”[Title/Abstract] OR “High-intensity interval” [Title/Abstract] OR “Yoga” [Title/Abstract] OR “Dance” [Title/Abstract] OR “Taichi” [Title/Abstract] OR “Baduanjin” [Title/Abstract] OR “Wuqinxi” [Title/Abstract] OR “Yijinjing” [Title/Abstract] OR “Walking” [Title/Abstract] OR “Physical and mental exercise” [Title/Abstract] #2“Depression”[Mesh] OR “Depressive disorder”[Title/Abstract] OR “Depressive symptom” [Title/Abstract] OR “Emotional depression” [Title/Abstract] OR “Depressive neurosis” [Title/Abstract] OR “Endogenous depression” [Title/Abstract] OR “Deurotic depression” [Title/Abstract] OR “Unipolar depression” [Title/Abstract] #3 “Attention” [Mesh] OR “Cognition” [Title/Abstract] OR “Cognitive performance” [Title/Abstract] #4 Randomized controlled trial[Publication Type] OR “Randomized” [Title/Abstract] OR “controlled”[Title/Abstract] OR “Trial” [Title/Abstract] #5 #1 AND #2 AND #3 AND #4
EMBASE	#1 “Exercise”[exp] OR “Aerobic exercise”[ab, ti] OR “Resistance exercise”[ab, ti] OR “High-intensity interval” [ab, ti] OR “Yoga” [ab, ti] OR “Dance” [ab, ti] OR “Taichi” [ab, ti] OR “Baduanjin” [ab, ti] OR “Wuqinxi” [ab, ti] OR “Yijinjing” [ab, ti] OR “Walking” [ab, ti] OR “Physical and mental exercise” [ab, ti] #2 “Depression”[exp] OR “Depressive disorder” [ab, ti] OR “Depressive symptom” [ab, ti] OR “Emotional depression” [ab, ti] OR “Depressive neurosis” [ab, ti] OR “Endogenous depression” [ab, ti] OR “Deurotic depression” [ab, ti] OR “Unipolar depression” [ab, ti] #3 “Attention” [exp] OR “Cognition” [ab, ti] OR “Cognitive performance” [ab, ti] #4 “Randomized controlled trial” [exp] OR “Randomized” [ab, ti] OR “Controlled” [ab, ti] OR “Trial” [ab, ti] #5 #1 AND #2 AND #3 AND #4
Web of Science	#1 TS = (“Exercise” OR “Aerobic exercise” OR “Resistance exercise” OR “High-intensity interval” OR “Yoga” OR “Dance” OR “Taichi” OR “Baduanjin” OR “Wuqinxi” OR “Yijinjing” OR “Walking” OR “Physical and mental exercise”) #2 TS = (“Depression” OR “Depressive disorder” OR “Depressive symptom” OR “Emotional depression” OR “Depressive neurosis” OR “Endogenous depression” OR “Deurotic depression” OR “Unipolar depression”) #3 TS = (“Cognition” OR “Cognitive performance” OR “Attention”) #4 TS = (“Randomized controlled trial” OR “Randomized” OR “Controlled” OR “Trial”) #5 #1 AND #2 AND #3 AND #4
CNKI	(运动exercise + 有氧运动aerobic exercise + 抗阻训练resistance training + 力量训练power training + 太极拳Tai Chi +瑜伽Yoga + 八段锦Baduanjin + 五禽戏Wuqinxi + 慢跑Jogging + 快走Speed Walking)AND (抑郁症Depression disease + 抑郁Depression)AND (认知功能cognition function + 注意力attention + 认知cognition)
WanFang	(运动exercise OR 有氧运动aerobic exercise OR 抗阻训练resistance training OR 力量训练power training OR 太极拳Tai Chi OR 瑜伽Yoga OR 八段锦Baduanjin OR 五禽戏Wuqinxi OR 慢跑Jogging OR 快走Speed Walking)AND(抑郁症Depression disease OR 抑郁Depression)AND(认知功能cognition function OR 注意力attention OR 认知cognition)

### 2.2 Literature inclusion criteria

The subjects were patients with depression, who met either the International Classification of Disease (ICD) or the Diagnostic and Statistical Manual of Mental Disorders (DSM) diagnostic criteria; The intervention content of the experimental group was exercise or exercise based on the control group, while the control group had no exercise intervention or conventional treatment, such as drug treatment, stretch relaxation, memory health education, etc.; Outcome Indicators: Testbatterie zur Aufmerksamkeitsprufung (TAP), Repeatable Battery for the Assessment of Neuropsychological Status (RBANS), Trail Making Test A (TMT), Stroop Interference(SI), D2-R, Digit Vigilance Test (DVT), Continuous Performance Task (CPT), Subtracting Serial Sevens (SSS), if the same literature has multiple assessment indicators for assessing attention, the indicators most frequently used in other studies will be used; The type of study was randomized controlled trial.

### 2.3 Exclusion criteria of literature

Studies were excluded if the intervention in the experimental group was not exercise or if the intervention in the control group involved exercise. Articles were also excluded if the full text was unavailable, or if data could not be obtained even after contacting the original authors. Studies were excluded if their outcome measures did not assess attention, or if the article type was a review or conference abstract. In addition, Chinese articles published in non-core journals were excluded. Studies that are not in Chinese or English will also be excluded.

### 2.4 Screening and data extraction of literature

Two researchers (HJ D and C L) independently screened the literature. First, the retrieved literature was imported into endnote X9 for summary and deduplication. Then, the titles and abstracts of the literature were read for preliminary screening. Finally, the full text of the literature after the preliminary screening was read for re-screening. If two researchers encounter differences, a third researcher (M Q) would discuss and make the final decision.

The information extracted by the two researchers (HJ D and C L) includes the author, year, sample size, age, country, intervention method, exercise frequency, exercise duration, exercise intensity, exercise cycle, and outcome indicators. In case of disagreement, a third researcher (M Q) would discuss and make the final decision. Among them, the exercise intensity is divided into low intensity, moderate intensity, and moderate to high intensity (Garber et al., 2011; Ren et al., 2023).

### 2.5 Quality evaluation

This study requires a methodological quality assessment of the included articles. Two researchers (HJ D and C L) used the modified version of the PEDro scale for quality evaluation (Ludyga et al., 2016). The scale includes 10 items: “Eligibility Criteria “, “Allocation of Randomization “, “Concealed Allocation “, “Similarity Baseline,” “Exercise Load Control,” “Assessor Blinding “, “More Than 85% Retention “, “Intention-to-Treat Analysis “, “Between-group Comparisons “and “Point and Variability Measures “. Each scoring dimension includes two rating criteria, assigned as either 0 points or 1 point. If the criteria are met, the score is 1 point, if the criteria are not met, the score is 0 points, and the total score is 10 points. Below 4 points means poor quality, 4–5 means medium quality, 6–8 good quality, 9–10 high quality (Cai et al., 2020). In case of disagreement, a third researcher (M Q) would discuss and make the final decision.

### 2.6 Evaluation of outcome evidence quality

GRADEpro software was used to evaluate the quality of outcome evidence. A total of five evaluation items included one by one [none (not degraded), severe (reduced by 1 level), and very severe limitations, inconsistencies, indirectness, imprecision, and publication bias, were evaluated (reduced by 2 levels)]. The level of evidence was divided into four levels: high quality, medium quality, low quality, and very low quality. The results were presented in the summary table of evidence. “High”: the result is very convinced that further research cannot change this result; “Medium”: the results are generally convinced, and further research may change the results; “Low”: the result is believed to be limited, and further research is probably to change the result; “Extremely low”: the results are almost unreliable, and further research is likely to change the results (Marino et al., 2021).

### 2.7 Statistical methods

Meta-analysis, subgroup analysis, and publication bias tests were performed using Stata 17.0. Due to variations in measurement methods and unit scales, the standardized mean difference was used to pool effect sizes. If the direction of measurement units was different, the average was multiplied by −1 to calculate the effect amount. Hedges’s g was used for effect quantity consolidation. Hedges’ g < 0.2 meant a small effect, 0.20–0.49 a mid-small effect, 0.50–0.79 a medium effect, and ≥ 0.8 a large effect (Jacob, 1992). Due to the small sample size in this study, Cohen’s d was not used; instead, Hedges’ g was employed. The formula for calculating the effect size Hedges’ g is as follows: $Var\left(\mathrm{Hedge}s^{\prime }\;g\right)=\frac{1}{n1}+\frac{1}{n2}+\frac{\left(\mathrm{Hedge}s^{\prime }\;g\right)2}{2\left(n1+n2\right)}$.And it was verified using the following formula: $\mathrm{Hedge}s^{\prime }g=\frac{M1-M2}{\sqrt{\frac{\left(n1-1\right)\left(S1\right)2+\left(n2-1\right)\left(S2\right)2}{n1+n2-2}}}$.I^2^ statistics were used for heterogeneity, and 75, 50, and 25% were the cut-off values of high, medium and low heterogeneity, respectively (Higgins et al., 2003). If medium and high heterogeneity should exist in the study, the random effect model would be used for effect quantity consolidation, and subgroup analysis and sensitivity analysis would be used to explore the source of heterogeneity, otherwise, the fixed effect model would be used.

## 3 Results

### 3.1 Literature search results

**Figure 1:**
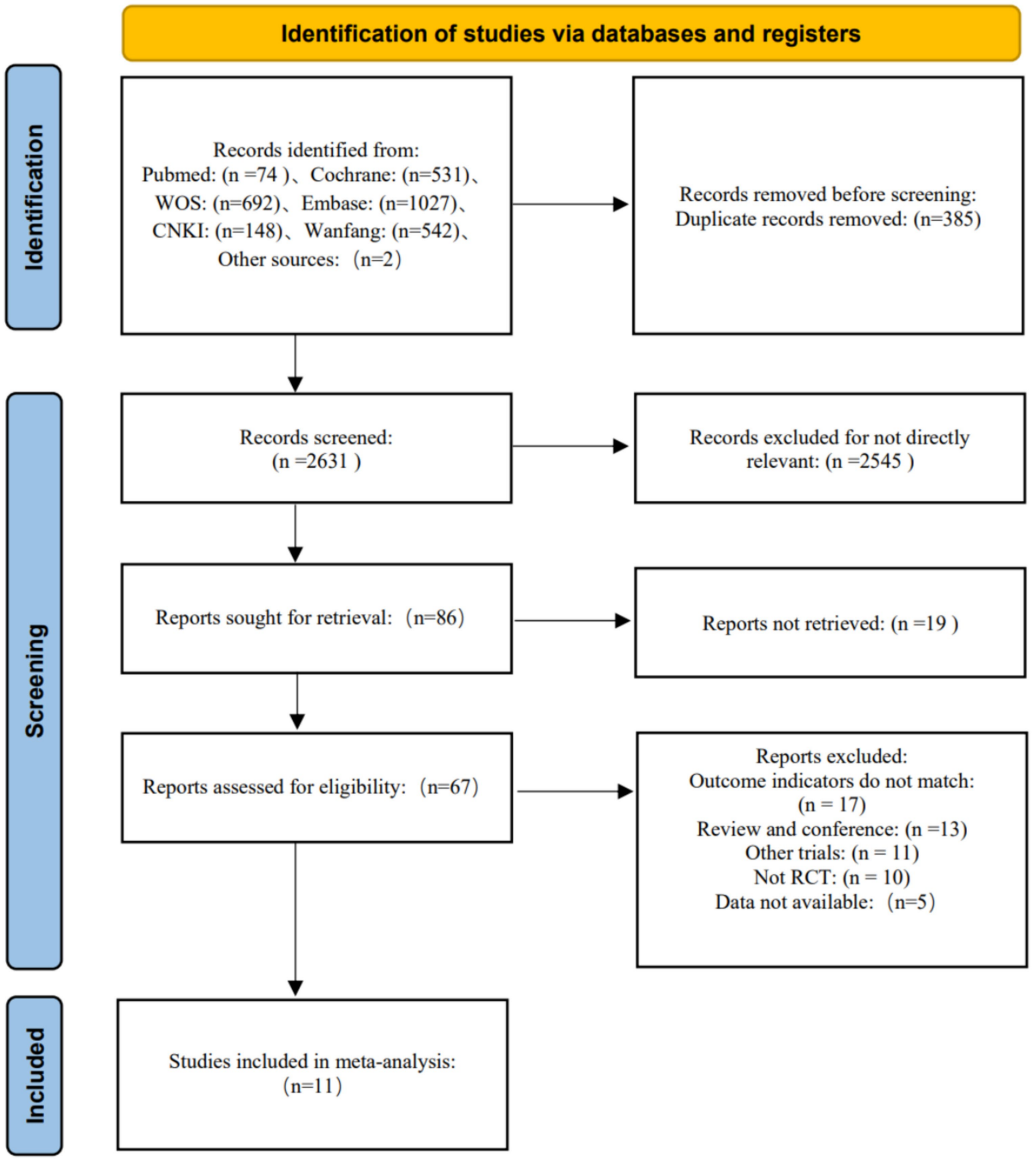
Flow chart of literature screening.

3,014 articles were retrieved from 6 databases and another 2 articles were obtained from other sources (Lavretsky et al., 2011; Luttenberger et al., 2015), Endnote X9 has 2,631 articles left after eliminating duplicate articles, and 86 articles left after reading the title and abstract, of which 19 articles could not be found, so 67 articles were read in full text. Among these, the outcome indicators of 17 articles were inconsistent, 13 were meetings or reviews, 11 were other trials, 10 were not RCT, and the data of 5 could not be extracted. Finally, 11 articles were included, as shown in Figure 1.

### 3.2 Characteristics of included literature

A total of 11 studies were included, including 4 from Asia (36.36%), 5 from Europe (45.45%), and 2 from North America (18.18%). There were 853 patients with depression, aged 23–79 years old. The exercise methods of the intervention group included aerobic exercise, yoga, Tai Chi, etc. the exercise frequency was 1–7 days/week, the exercise duration was 30–180 min, the exercise cycle was 3–16 weeks, and the exercise intensity was divided into low intensity, moderate intensity, and moderate-to-vigorous intensity. Outcome indicators include TAP, DVT, DSB, and other indicators, as shown in Table 3.

**Table 3:** Basic characteristics of included studies.

Included literature	Country	Number(E/C)	Age(E/C)	Type of Intervention	Motion frequency	Length	Intensity	Length of intervention	Outcome measure
Buschert et al. (2019)	Germany	18/20	47.27 ± 6.84/47.47 ± 8.47	E: Aerobic exercise;C: Occupational or art therapy	2-3d/wk	30 min	85%HRmax/Moderate-to-Vigorous	3-4wk	TAP
Chan et al. (2012)	China	17/16	47.06 ± 9.54/45.44 ± 2.59	E: Chanwuyi;C: Waiting control	1d/wk	90 min	Low	10 wk	DVT
Imboden et al. (2020)	Switzerland	22/20	41.3 ± 9.2/38.3 ± 13.4	E: Aerobic exercise; C: Stretching	3 d/wk	45 min	60-75%HRmax/Moderate	6 wk	TAP
Krogh et al. (2009)	Denmark	55/55/55	41.9 ± 8.7(resistance)/38.1 ± 9(aerobic)/36.7 ± 8.7	E: Strength training/Aerobic exercise;C: Relaxation	2 d/wk	90 min	50-75%RM、70-89%HRmax/Moderate-to-Vigorous	16 wk	SSS
Krogh et al. (2012)	Denmark	56/59	39.7 ± 11.3/43.4 ± 11.2	E: Aerobic exercise; C: Stretching	3d/wk	45 min	65-80%VO_2_max/ Moderate-to-Vigorous	12 wk	SSS
Sharma et al. (2006)	India	15/15	31.87 ± 8.78/31.67 ± 8.46	E: Yoga; C: Same hand postures as yoga group (no meditation)	3 d/wk	30 min	Low	8 wk	TMT
Chen et al. (2021)	China	63/62	30.3 ± 7.5/32.7 ± 6.5	E: Aerobic exercise; C: Antidepressant	≥3 d/wk	30–60 min	64-76%HRmax/Moderate	8, 16 wk	CPT
Zheng et al. (2019)	China	30/30	37.5 ± 9.12/35.17 ± 5.93	E: Aerobic exercise; C: Conventional Therapy	5 d/wk	60 min	Moderate	4 wk	RBANS
Lavretsky et al. (2011)	USA	36/37	69.1 ± 7.0/72.0 ± 7.4	E: Tai Chi; C: Health education	1 d/wk	120 min	Low	10 wk	TMT
Lavretsky et al. (2022)	USA	62/63	69.2 ± 6.9/69.4 ± 6.2	E: Tai Chi; C: Health education	1 d/wk	60 min	Low	12 wk	TMT + SI
Luttenberger et al. (2015)	Germany	22/25	42.71 ± 11.88/44.96 ± 12.08	E: Indoor rock climbing; C:Waiting control	1 d/wk	180 min	Low	8 wk	D2-R

E for Experimental group; C for Control group; wk for week; USA for United States of America; HR max for Maximal Heart Rate, VO_2_ max for Maximal Oxygen Uptake; RM for Repetition Maximum; min for Minutes wk for week; USA for United States of America; HR max for Maximal Heart Rate,VO_2_ max for Maximal Oxygen Uptake; RM for Repetition Maximum; min for Minutes.

### 3.3 Literature quality evaluation

The 11 included studies all achieved the “eligibility criteria,” “random assignment,” “baseline similarity,” “statistical analysis between groups” and “point measurement and variance value.” Among these, two studies showed the “allocation concealment” method, six studies showed how to carry out “exercise load control,” four studies did not have the “blinding of result evaluation,” six studies did not achieve the “withdrawal rate<15% and four studies achieved the “ITT intention to treat analysis.” The total score was 5–9, with an average of 7.18, as shown in Table 4.

**Table 4:** Quality evaluation table of methodology of included literature.

Included literature	1	2	3	4	5	6	7	8	9	10	TS
Sharma et al. (2006)	1	1	0	1	0	1	1	1	1	1	8
Imboden et al. (2020)	1	1	0	1	1	1	0	0	1	1	7
Lavretsky et al. (2011)	1	1	0	1	0	1	1	0	1	1	7
Lavretsky et al. (2022)	1	1	0	1	0	1	0	0	1	1	6
Buschert et al. (2019)	1	1	0	1	1	0	0	0	1	1	6
Luttenberger et al. (2015)	1	1	0	1	0	0	0	0	1	1	5
Krogh et al. (2009)	1	1	0	1	1	1	1	0	1	1	8
Krogh et al. (2012)	1	1	1	1	1	1	0	1	1	1	9
Zheng et al. (2019)	1	1	0	1	1	0	1	1	1	1	8
Chen et al. (2021)	1	1	0	1	1	0	1	1	1	1	8
Chan et al. (2012)	1	1	1	1	0	1	0	0	1	1	7

1: Eligibility criteria; 2: allocation of randomization; 3: allocation concealment; 4: similarity baseline; 5: Exercise load control; 6:Assessor blinding; 7: more than 85% retention; 8: intention-to-treat analysis; 9: between-group comparisons; 10: point and variability measures; TS: total score.

### 3.4 Meta-analysis results

**Figure 2:**
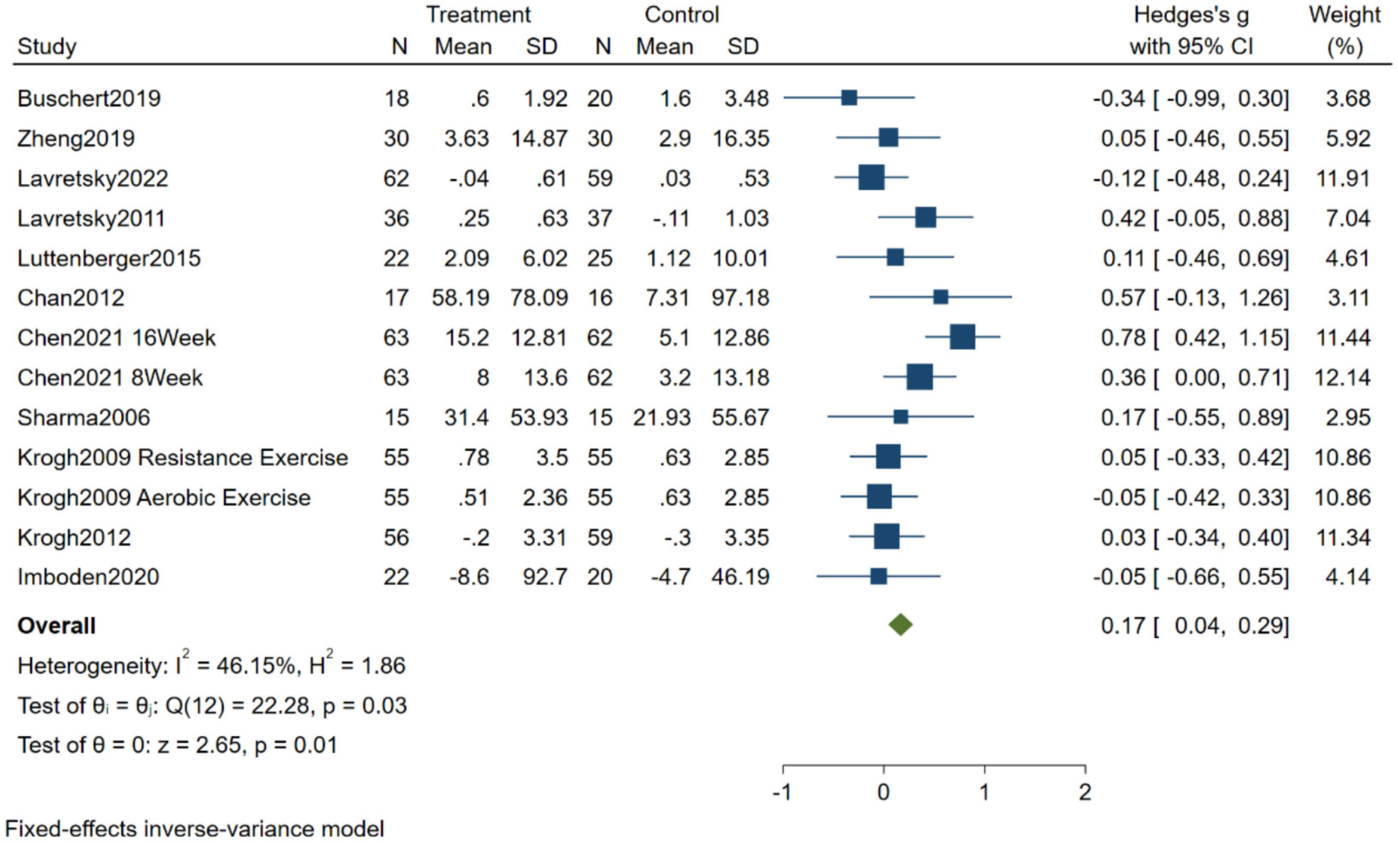
Forest diagram of the effect of exercise on attention intervention in patients with depression.

A total of 13 effect sizes and 924 patients were conducted to study the effect of exercise on attention intervention in patients with depression. The heterogeneity test results showed that I^2^ = 46.15%, and there was low heterogeneity between the studies, therefore the fixed effect model was used for analysis. Meta-analysis showed that the hedge’s g = 0.17, 95% CI (0.04, 0.29), *p* = 0.01, has statistical significance, as shown in Figure 2.

### 3.5 Sensitivity analysis

**Figure 3:**
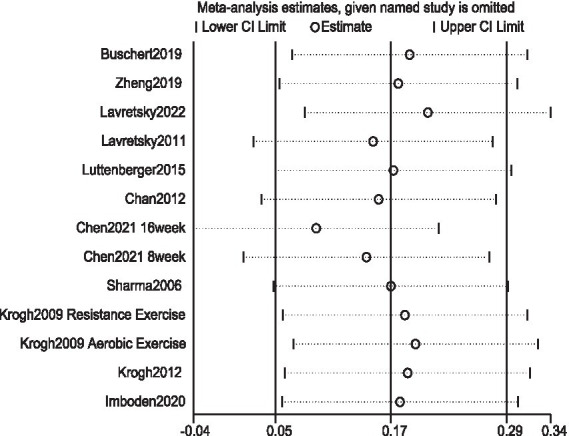
Sensitivity analysis of the effect of exercise on attention intervention in patients with depression.

To explore the source of heterogeneity, each study was excluded one after another to find whether heterogeneity was caused by certain individual studies. The results showed that the effect quantities were all in 95% CI, and the research results were relatively stable, as shown in Figure 3.

### 3.6 Subgroup analysis

To further explore the source of heterogeneity, subgroup analysis was carried out on various elements of sports. Due to the commonness of exercise frequency, subgroup analysis could not be conducted. Therefore, subgroup analysis was only conducted on exercise form, exercise duration, exercise intensity, and exercise cycle, as shown in Table 5.

**Table 5:** Subgroup analysis of the effect of exercise on attention intervention in patients with depression.

Adjusting variables	Q, P	I^2^	N	Hedge’s g, 95%CI	*P*
Intervention type	0.52, 0.77				
Aerobic exercise		64.33%	7	0.20, (0.04,0.36)	0.01
Chinese traditional sports		58.70%	3	0.15, (−0.12,0.41)	0.27
Others		0.00%	3	0.08, (−0.20,0.37)	0.57
Duration of exercise (minutes)	1.10, 0.58				
30–60		61.60%	8	0.18, (0.03,0.34)	0.02
61–90		13.58%	3	0.07, (−0.18,0.32)	0.57
>90		0.00%	2	0.30, (−0.06,0.66)	0.11
Cycle of exercise (week)	0.19, 0.66				
3–12		53.80%	10	0.19, (0.04,0.34)	0.02
13–16		23.40%	3	0.13, (−0.08,0.34)	0.24
Intensity of exercise	7.94, 0.02				
Low		17.67%	5	0.14, (−0.08,0.37)	0.21
Moderate		63.85%	4	0.40, (0.18,0.61)	0.00
Moderate-to-vigorous		0.00%	4	−0.03, (−0.23,0.18)	0.81

The form of exercise did not play a regulatory role (Q = 0.52, *p* = 0.77). The exercise forms were divided into “aerobic exercise,” “Chinese traditional exercise” and “other” groups. Only the “aerobic exercise” group had a significant effect, Hedge’s *g* = 0.20, *p* = 0.01. There was no statistical significance in other groups.

Exercise duration did not play a regulatory role (*Q* = 1.10, *p* = 0.58). The exercise duration was divided into “30–60,” “61–90” and “>90” minutes groups. Only in the “30–60” minute group, there was a significant effect, Hedge’s *g* = 0.18, *p* = 0.02. There was no statistical significance in other groups.

The exercise cycle did not play a regulatory role (*Q* = 0.19, *p* = 0.66). The exercise cycle was divided into “3–12” and “13–16” weeks groups. Only the “3–12” week group showed a significant effect, Hedge’s *g* = 0.19, p = 0.02. There was no statistical significance in other groups.

Exercise intensity played a regulatory role (*Q* = 7.94, p = 0.02). The exercise intensity was divided into “low intensity,” “moderate intensity” and “moderate to vigorous intensity” groups. Only in the “moderate intensity” group, the effect quantity was statistically significant, with Hedge’s *g* = 0.40, *p* = 0.00. There was no statistical significance in other groups.

### 3.7 Publication bias

**Figure 4:**
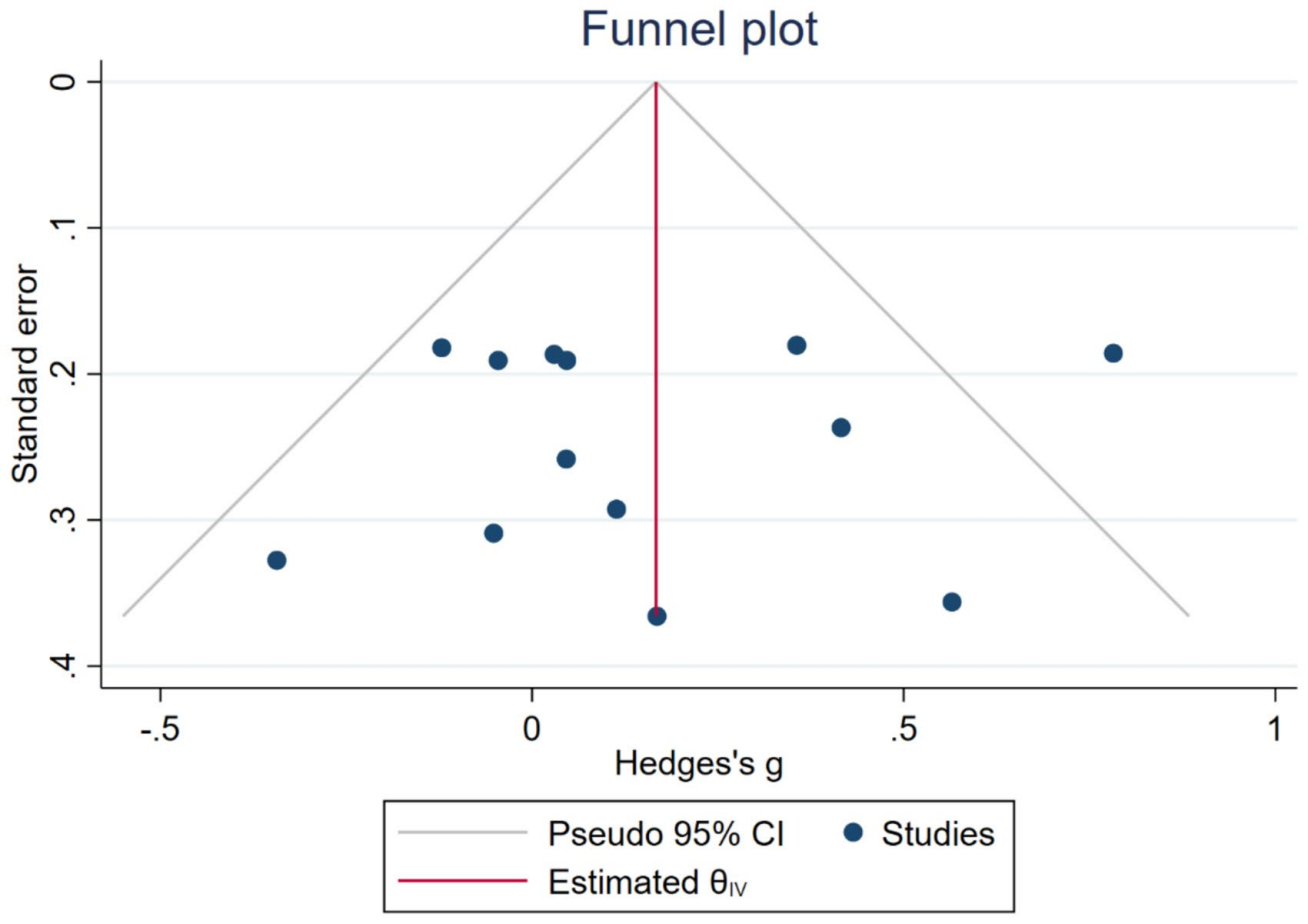
Funnel chart of the effect of exercise on attention intervention in patients with depression.

The funnel plot shows that both sides are symmetrical, and the Egger test shows that *z* = −0.49, P >| Z | = 0.62, indicating that there is no potential publication bias, language bias, and small sample bias in this study, as shown in Figure 4.

### 3.8 GRADEpro evidence quality evaluation results

GRADEpro was used to evaluate the results, and it was found that GRADE was low, as shown in Table 6.

**Table 6:** Level of evidence for outcome indicators.

Outcome	RCTs	Evaluation of evidence quality level				Sample Size			Relative effect (95%CI)	Level
		Study limitation	Inconsistency	Indirectness	Imprecision	Publication bias	Experimental group	control group		
Attention	11	Downgrade 1 Level^A^	Downgrade 1 Level^B^	Not Downgraded	Not Downgraded	Not Downgraded	451	398	0.17, 95%CI:0.04, 0.29	Low

^A for Some studies did not realize allocation concealment and evaluation blinding; B for Some studies did not distinguish the degree of depression in patients with depression.^

## 4 Discussion and analysis

Currently, the effects of exercise on attention in patients with depression remain controversial. Building upon previous research, this study included a larger number of randomized controlled trials and employed a meta-analysis to systematically and comprehensively evaluate the effects of exercise on attention in patients with depression. The low evidence level suggests that exercise can improve attention in patients with depression, and for the first time, it was identified that exercise intensity is a moderating factor in the effect of exercise interventions on attention in these patients. This study utilized tasks such as the TMT and the Stroop test to assess attention in individuals with depression. However, the TMT and Stroop test may not be purely attention-specific assessment tools. For instance, the TMT evaluates not only attention but also information processing speed and executive function, while the Stroop test assesses inhibitory control and executive function in addition to attention (Lavretsky et al., 2022). This limitation may contribute to the discrepancies between the findings of this study and those of previous research.

The results showed that exercise could improve the attention of patients with depression, and there was a small effect size (Hedge’s *g* = 0.17, *p* = 0.01). Research has found that patients with depression exhibit greater alpha power in the left frontal lobe compared to the right frontal lobe, whereas healthy individuals show the opposite pattern. This suggests that abnormalities exist in both the structure and function of the brain in individuals with depression (Wang et al., 2019). Given that the frontal lobe is closely associated with cognitive functions such as attention, other studies have found a significant positive correlation between frontal lobe power values and scores on cognitive tasks. This further suggests that changes in the frontal lobe may lead to abnormalities in cognitive function (Brzezicka et al., 2017). Exercise can improve the attention of patients with depression, which may be related to the improvement of brain structure and function. Animal studies have shown that multi-component aerobic exercise can enhance the activation of the medial agranular cortex, dorsolateral prefrontal cortex, medial prefrontal cortex, and anterior cingulate cortex and enhance the functional connection between the anterior limbic system and cerebellum, as well as between motor cortex and sensory-motor cortex (Wang et al., 2015). In addition, the improvement of attention in patients with depression through exercise can be explained by the “Energetic-resource model” and “Ego depletion” theories. During exercise, cognitive resources are consumed as individuals must remember movements, technical skills, or observe the exercise environment. After exercise, participants experience relaxation or fatigue, and during rest, their cognitive resources are replenished, thereby facilitating the improvement of attention (Schumann et al., 2022; Steghaus and Poth, 2024). This research results are inconsistent with the previous results. The systematic review of Brondino et al. (2017) and Sun et al. (2018) showed that exercise did not improve the attention of patients with depression, which may be attributed to the fact that the previous studies included 6 and 7 effect sizes, while this study included 13, avoiding small sample bias to a certain extent; secondly, the evaluation tools of the outcome indicators included in the study are different, and different measurement tools have differences in the evaluation of each dimension of attention. For example, the connection test type A and the number symbol test are timed tests, which reflect the speed of visual perception movement in attention function. The continuous work task mainly measures the ability to maintain attention, and the number span reverse test mainly measures the ability to change attention (Zhao et al., 2007).

Our study found that exercise intensity is a regulatory variable that affects the effect of exercise on attention intervention in patients with depression. Only the “moderate intensity” group had the moderate effect (Hedge’s *g* = 0.40, *p* = 0.00). Compared with low-intensity and medium-intensity, moderate-intensity exercise was easier to accept and adhere to, and long-term moderate-intensity exercise could get better results (Gan and Zhong, 2023). According to the wake-up theory hypothesis, moderate-intensity exercise is more conducive to cognitive performance than low-intensity and high-intensity exercise, which may be because moderate-intensity exercise is more conducive to the release of catecholamines and increases the biological wake-up of the central nervous system, thus improving the efficiency of cognitive resource allocation (Kamijo et al., 2004). At present, there is no study to explore the effect of exercise intensity on the attention of patients with depression, but there are similar results in other studies of cognitive function. Ren et al. found that moderate intensity had the best effect on the improvement of executive function in patients with depression (Ren et al., 2023). Jiang et al. found that moderate-intensity aerobic exercise can improve visual learning, memory, and executive function in patients with depression, but with the increase of exercise intensity to a high level, aerobic exercise can only improve visual learning and memory function, but not executive function (Jiang et al., 2022).

Our study also found that exercise form, exercise duration, and exercise cycle are not regulatory variables. In the form of exercise, only “aerobic exercise” can improve attention, with a small effect size (Hedge’s *g* = 0.20, *p* = 0.01), while “Chinese traditional exercise” and “other” groups do not affect attention. The effect of exercise forms on cognitive function is specific, and may be related to the different physiological bases of different exercise forms. Aerobic exercise is more conducive to increasing cerebral blood perfusion, improving cerebral blood oxygen levels (Guo and Wang, 2020), and providing sufficient energy for neuronal activity (Vogiatzis et al., 2011). Long-term aerobic exercise can promote the activity level of the frontal lobe, bilateral frontotemporal lobe, anterior cingulate cortex, and other brain regions, which is conducive to the improvement of executive functions such as inhibition related to attention (Kramer et al., 1999). Resistance exercise is beneficial to the improvement of peripheral brain-derived neurotrophic factors and memory (Marinus et al., 2019; Yu, 2020). Other studies also share this conclusion: Cai and other experts agree that aerobic exercise and resistance exercise have a better effect on improving executive function, while traditional Chinese sports have a better effect on improving overall cognitive function (Cai et al., 2021). In the exercise duration, only the “30–60” minute group can improve attention, and there is a small effect (Hedge’s *g* = 0.18, *p* = 0.02). The “61–90” minute group and the “>90” minute group have no improvement in attention, which may be because long-term continuous exercise may over-activate the premotor and auxiliary motor areas of the brain, damaging cognitive performance, and long-term exercise can increase fatigue, dehydration, which is not conducive to cognitive performance (Audiffren and André, 2015; Loprinzi and Kane, 2015). In the exercise cycle, the “3–12” week group can improve attention, with a small effect (hedge’s *g* = 0.19, *p* = 0.02), while the “13–16” week group has no improvement in attention. This may be because the population included in this study are patients with depression, and an early study showed that long-term exercise, no matter how long it lasts each time, may cause a certain degree of psychological damage to patients with depression (Langosch, 1988). This may further aggravate the patient’s attention loss. However, many subgroups did not demonstrate statistical significance, and these findings should be interpreted with caution. This may be attributed to the limited number of included studies or small effect sizes, which could have reduced the statistical power (Cheung, 2014).

This study included only randomized controlled trials, while non-randomized controlled trials were not considered, which may affect the reliability of the results. Although all the literature included in this study were randomized controlled trials, most of them were designed to achieve allocation concealment, evaluation blinding, and researcher blinding, which increased the additional risk of misleading results and affected the reliability of the results. Although the population of this study is depression patients, most of the studies included did not provide a more detailed classification of depression patients, which may also lead to inconsistent results. This study only examined the overall effect of exercise on attention in patients with depression, without categorizing attention or further exploring the components of attention. This study utilized a diverse range of assessment tools to evaluate attention in individuals with depression. However, some of these tools are not exclusively designed for attention assessment. Therefore, future research should be based on randomized controlled trial design, to increase allocation concealment, blinding by researchers, and evaluation blinding as much as possible to improve methodological quality. It is also necessary to have a clearer classification of depression patients and develop more refined exercise prescriptions for individuals with different degrees of depression. Future studies should also incorporate a detailed categorization of attention to further elucidate the effects of exercise on attentional function in individuals with depression. Additionally, standardizing attention assessment tools as much as possible is essential to minimize heterogeneity arising from variations in measurement instruments. Furthermore, future research may consider including studies with diverse experimental designs to enhance the reliability of the findings. Finally, due to the limited number of included studies, a detailed classification of exercise types was not possible; therefore, the results should be interpreted with caution. Future research should consider the heterogeneity of different exercise types and adopt more rigorous randomized controlled trials with larger sample sizes to compare the effects and differences of various exercise modalities on attention in patients with depression.

## 5 Conclusion

In conclusion, exercise can improve the attention of patients with depression, and moderate-intensity exercise has the best effect, while exercise form, exercise duration, and exercise cycle do not affect the intervention effect. In future studies, a more rigorous methodological design should be adopted and more large sample, multi-center clinical randomized controlled studies should be included to prove the clinical efficacy of exercise on the attention of patients with depression.

## Footnotes

^1^ https://www.crd.york.ac.uk/prospero/
